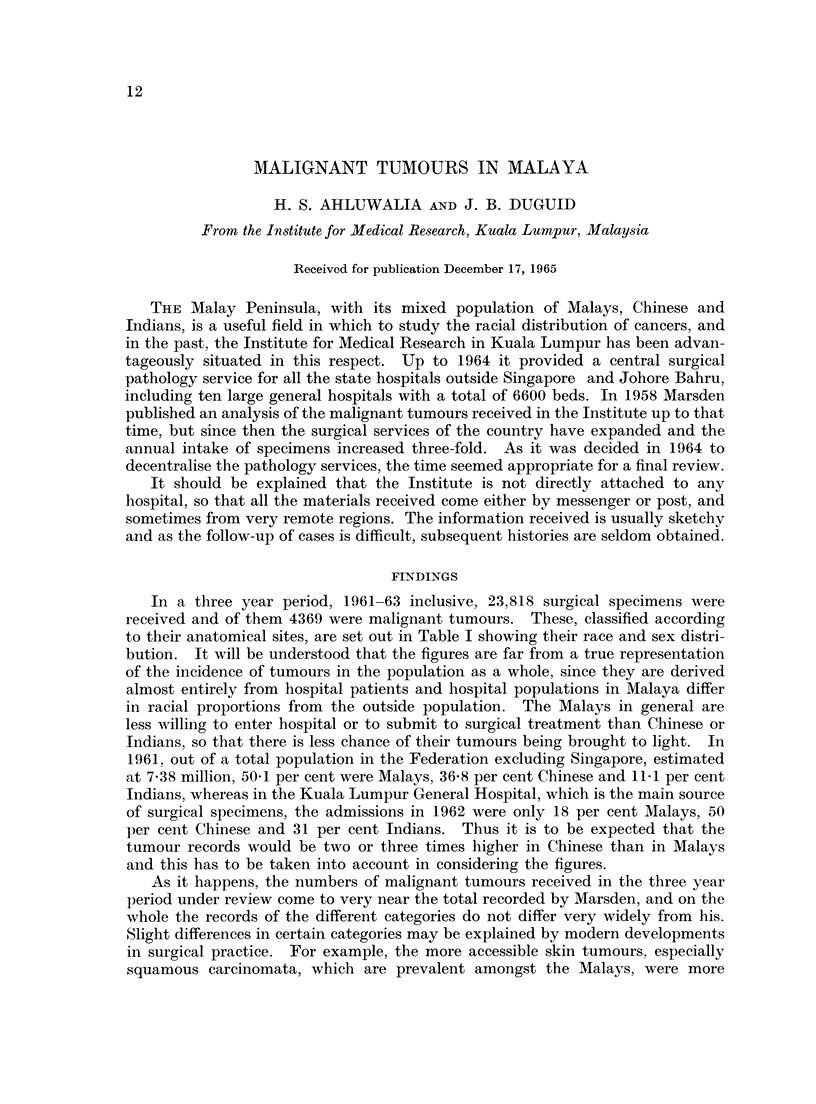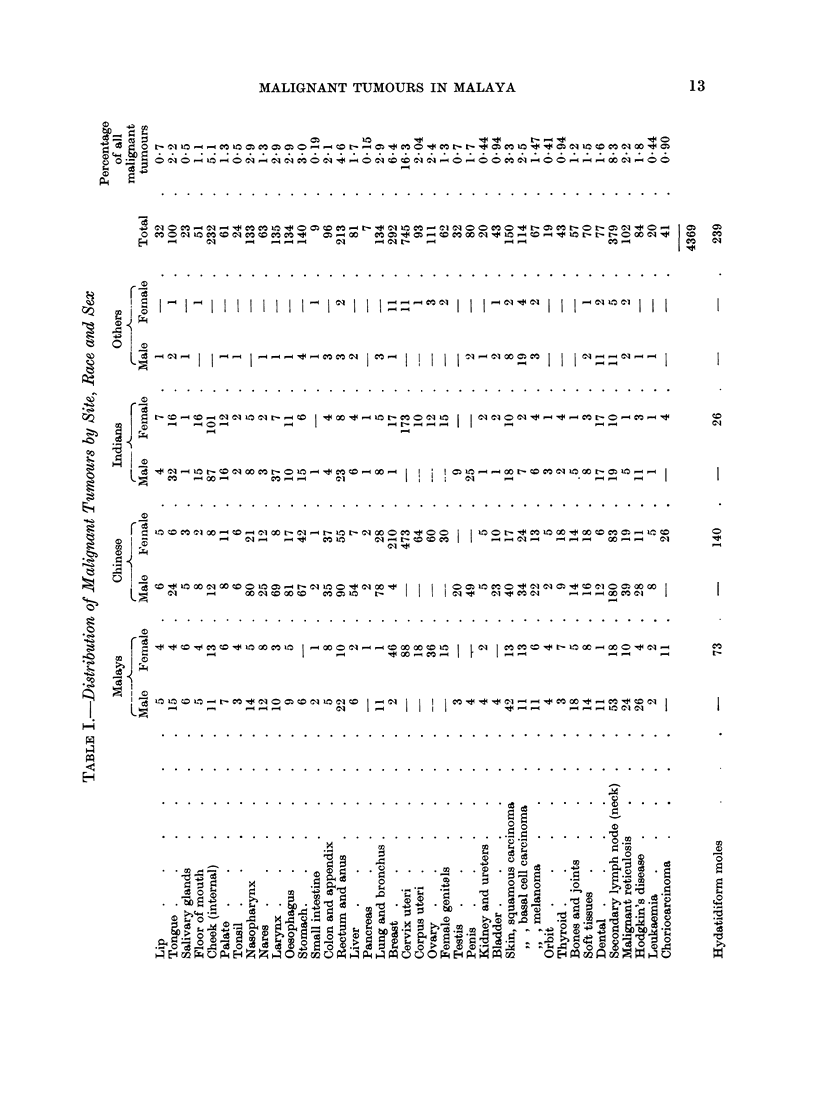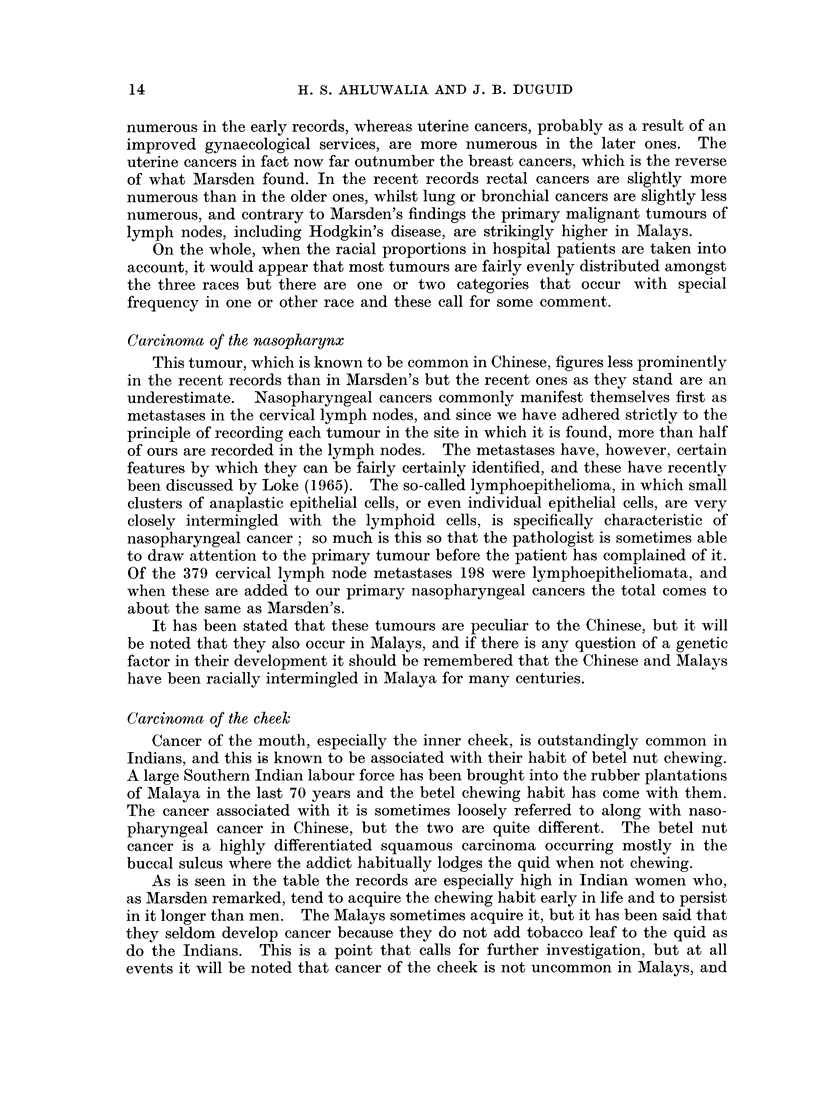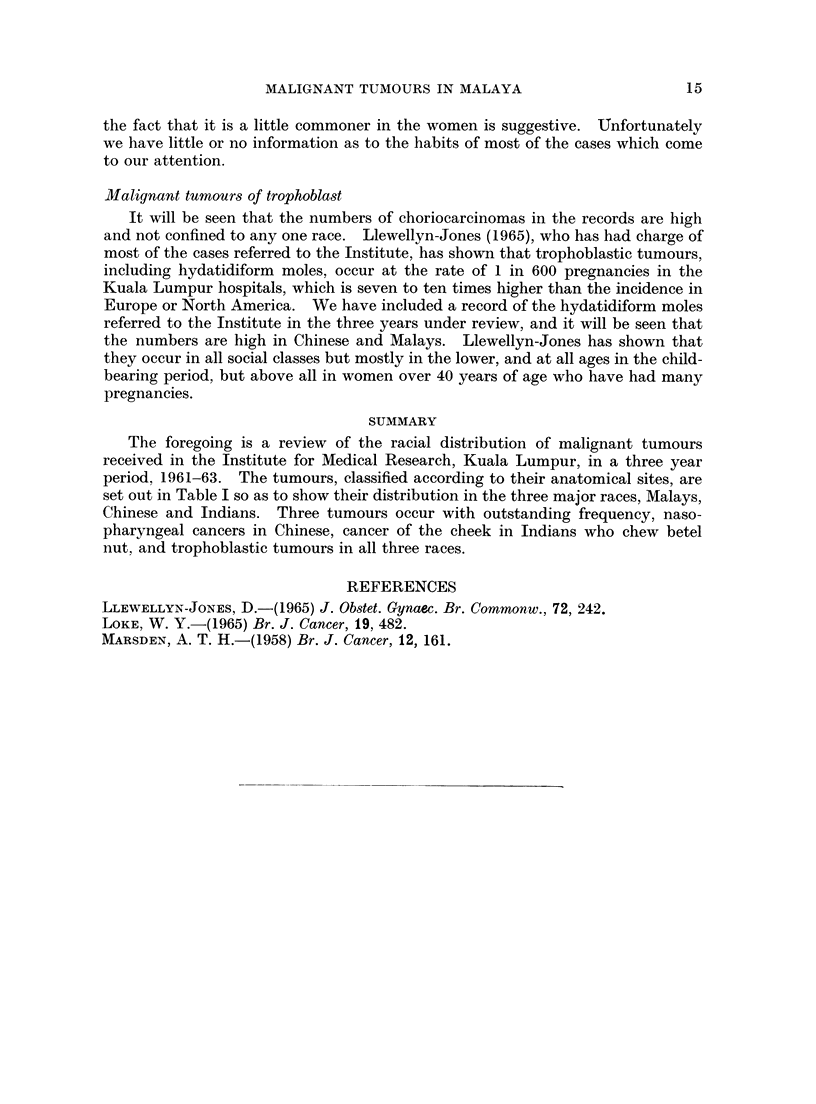# Malignant tumours in Malaya.

**DOI:** 10.1038/bjc.1966.2

**Published:** 1966-03

**Authors:** H. S. Ahluwalia, J. B. Duguid


### MALIGNANT TUMOURS IN MALAYA

H. S. AHLUWALIA AND J. B. DUGUID

*From the Institute for Medical Research, Kuala Lumpur, Malaysia*

Received for publication December 17, 1965

THE Malay Peninsula, with its mixed population of Malays, Chinese and Indians, is a useful field in which to study the racial distribution of cancers, and in the past, the Institute for Medical Research in Kuala Lumpur has been advantageously situated in this respect. Up to 1964 it provided a central surgical pathology service for all the state hospitals outside Singapore and Johore Bahru, including ten large general hospitals with a total of 6600 beds. In 1958 Marsden published an analysis of the malignant tumours received in the Institute up to that time, but since then the surgical services of the country have expanded and the annual intake of specimens increased three-fold. As it was decided in 1964 to decentralise the pathology services, the time seemed appropriate for a final review.

It should be explained that the Institute is not directly attached to any hospital, so that all the materials received come either by messenger or post, and sometimes from very remote regions. The information received is usually sketchy and as the follow-up of cases is difficult, subsequent histories are seldom obtained.

### FINDINGS

In a three year period, 1961–63 inclusive, 23,818 surgical specimens were received and of them 4369 were malignant tumours. These, classified according to their anatomical sites, are set out in Table I showing their race and sex distribution. It will be understood that the figures are far from a true representation of the incidence of tumours in the population as a whole, since they are derived almost entirely from hospital patients and hospital populations in Malaya differ in racial proportions from the outside population. The Malays in general are less willing to enter hospital or to submit to surgical treatment than Chinese or Indians, so that there is less chance of their tumours being brought to light. In 1961, out of a total population in the Federation excluding Singapore, estimated at 7·38 million, 50·1 per cent were Malays, 36·8 per cent Chinese and 11·1 per cent Indians, whereas in the Kuala Lumpur General Hospital, which is the main source of surgical specimens, the admissions in 1962 were only 18 per cent Malays, 50 per cent Chinese and 31 per cent Indians. Thus it is to be expected that the tumour records would be two or three times higher in Chinese than in Malays and this has to be taken into account in considering the figures.

As it happens, the numbers of malignant tumours received in the three year period under review come to very near the total recorded by Marsden, and on the whole the records of the different categories do not differ very widely from his. Slight differences in certain categories may be explained by modern developments in surgical practice. For example, the more accessible skin tumours, especially squamous carcinomata, which are prevalent amongst the Malays, were more

TABLE I.—*Distribution of Malignant Tumours by Site, Race and Sex*

| | Malays | | Chinese | | Indians | | Others | | | Percentage of all malignant tumours |
|---|---|---|---|---|---|---|---|---|---|---|
| | Male | Female | Male | Female | Male | Female | Male | Female | Total | |
| Lip | 5 | 4 | 6 | 5 | 4 | 7 | 1 | — | 32 | 0·7 |
| Tongue | 15 | 4 | 24 | 6 | 32 | 16 | 2 | 1 | 100 | 2·2 |
| Salivary glands | 6 | 6 | 5 | 3 | 1 | 1 | 1 | — | 23 | 0·5 |
| Floor of mouth | 5 | 4 | 8 | 2 | 15 | 16 | — | 1 | 51 | 1.1 |
| Cheek (internal) | 11 | 13 | 12 | 8 | 87 | 101 | — | — | 232 | 5.1 |
| Palate | 7 | 6 | 8 | 11 | 16 | 12 | 1 | — | 61 | 1.3 |
| Tonsil | 3 | 4 | 6 | 6 | 2 | 2 | 1 | — | 24 | 0·5 |
| Nasopharynx | 14 | 5 | 80 | 21 | 8 | 5 | — | — | 133 | 2·9 |
| Nares | 12 | 8 | 25 | 12 | 3 | 2 | 1 | — | 63 | 1·3 |
| Larynx | 10 | 3 | 69 | 8 | 37 | 7 | 1 | — | 135 | 2·9 |
| Oesophagus | 9 | 5 | 81 | 17 | 10 | 11 | 1 | — | 134 | 2·9 |
| Stomach | 6 | — | 67 | 42 | 15 | 6 | 4 | — | 140 | 3·0 |
| Small intestine | 2 | 1 | 2 | 1 | 1 | — | 1 | 1 | 9 | 0·19 |
| Colon and appendix | 5 | 8 | 35 | 37 | 4 | 4 | 3 | — | 96 | 2·1 |
| Rectum and anus | 22 | 10 | 90 | 55 | 23 | 8 | 3 | 2 | 213 | 4·6 |
| Liver | 6 | 2 | 54 | 7 | 6 | 4 | 2 | — | 81 | 1·7 |
| Pancreas | — | 1 | 2 | 2 | 1 | 1 | — | — | 7 | 0·15 |
| Lung and bronchus | 11 | 1 | 78 | 28 | 8 | 5 | 3 | — | 134 | 2·9 |
| Breast | 2 | 46 | 4 | 210 | 1 | 17 | 1 | 11 | 292 | 6·4 |
| Cervix uteri | — | 88 | — | 473 | — | 173 | — | 11 | 745 | 16·3 |
| Corpus uteri | — | 18 | — | 64 | — | 10 | — | 1 | 93 | 2·04 |
| Ovary | — | 36 | — | 60 | — | 12 | — | 3 | 111 | 2·4 |
| Female genitals | — | 15 | — | 30 | — | 15 | — | 2 | 62 | 1·3 |
| Testis | 3 | — | 20 | — | 9 | — | — | — | 32 | 0·7 |
| Penis | 4 | — | 49 | — | 25 | — | 2 | — | 80 | 1·7 |
| Kidney and ureters | 4 | 2 | 5 | 5 | 1 | 2 | 1 | — | 20 | 0·44 |
| Bladder | 4 | — | 23 | 10 | 1 | 2 | 2 | 1 | 43 | 0·94 |
| Skin, squamous carcinoma | 42 | 13 | 40 | 17 | 18 | 10 | 8 | 2 | 150 | 3·3 |
| „ , basal cell carcinoma | 11 | 13 | 34 | 24 | 7 | 2 | 19 | 4 | 114 | 2·5 |
| „ , melanoma | 11 | 6 | 22 | 13 | 6 | 4 | 3 | 2 | 67 | 1·47 |
| Orbit | 4 | 4 | 2 | 5 | 3 | 1 | — | — | 19 | 0·41 |
| Thyroid | 3 | 7 | 9 | 18 | 2 | 4 | — | — | 43 | 0·94 |
| Bones and joints | 18 | 5 | 14 | 14 | 5 | 1 | — | — | 57 | 1·2 |
| Soft tissues | 14 | 8 | 16 | 18 | 8 | 3 | 2 | 1 | 70 | 1·5 |
| Dental | 11 | 1 | 12 | 6 | 17 | 17 | 11 | 2 | 77 | 1·6 |
| Secondary lymph node (neck) | 53 | 18 | 180 | 83 | 19 | 10 | 11 | 5 | 379 | 8·3 |
| Malignant reticulosis | 24 | 10 | 39 | 19 | 5 | 1 | 2 | 2 | 102 | 2·2 |
| Hodgkin's disease | 26 | 4 | 28 | 11 | 11 | 3 | 1 | — | 84 | 1·8 |
| Leukaemia | 2 | 2 | 8 | 5 | 1 | 1 | 1 | — | 20 | 0·44 |
| Choriocarcinoma | — | 11 | — | 26 | — | 4 | — | — | 41 | 0·90 |
| | | | | | | | | | 4369 | |
| Hydatidiform moles | — | 73 | — | 140 | — | 26 | — | — | 239 | |

numerous in the early records, whereas uterine cancers, probably as a result of an improved gynaecological services, are more numerous in the later ones. The uterine cancers in fact now far outnumber the breast cancers, which is the reverse of what Marsden found. In the recent records rectal cancers are slightly more numerous than in the older ones, whilst lung or bronchial cancers are slightly less numerous, and contrary to Marsden's findings the primary malignant tumours of lymph nodes, including Hodgkin's disease, are strikingly higher in Malays.

On the whole, when the racial proportions in hospital patients are taken into account, it would appear that most tumours are fairly evenly distributed amongst the three races but there are one or two categories that occur with special frequency in one or other race and these call for some comment.

*Carcinoma of the nasopharynx*

This tumour, which is known to be common in Chinese, figures less prominently in the recent records than in Marsden's but the recent ones as they stand are an underestimate. Nasopharyngeal cancers commonly manifest themselves first as metastases in the cervical lymph nodes, and since we have adhered strictly to the principle of recording each tumour in the site in which it is found, more than half of ours are recorded in the lymph nodes. The metastases have, however, certain features by which they can be fairly certainly identified, and these have recently been discussed by Loke (1965). The so-called lymphoepithelioma, in which small clusters of anaplastic epithelial cells, or even individual epithelial cells, are very closely intermingled with the lymphoid cells, is specifically characteristic of nasopharyngeal cancer ; so much is this so that the pathologist is sometimes able to draw attention to the primary tumour before the patient has complained of it. Of the 379 cervical lymph node metastases 198 were lymphoepitheliomata, and when these are added to our primary nasopharyngeal cancers the total comes to about the same as Marsden's.

It has been stated that these tumours are peculiar to the Chinese, but it will be noted that they also occur in Malays, and if there is any question of a genetic factor in their development it should be remembered that the Chinese and Malays have been racially intermingled in Malaya for many centuries.

*Carcinoma of the cheek*

Cancer of the mouth, especially the inner cheek, is outstandingly common in Indians, and this is known to be associated with their habit of betel nut chewing. A large Southern Indian labour force has been brought into the rubber plantations of Malaya in the last 70 years and the betel chewing habit has come with them. The cancer associated with it is sometimes loosely referred to along with nasopharyngeal cancer in Chinese, but the two are quite different. The betel nut cancer is a highly differentiated squamous carcinoma occurring mostly in the buccal sulcus where the addict habitually lodges the quid when not chewing.

As is seen in the table the records are especially high in Indian women who, as Marsden remarked, tend to acquire the chewing habit early in life and to persist in it longer than men. The Malays sometimes acquire it, but it has been said that they seldom develop cancer because they do not add tobacco leaf to the quid as do the Indians. This is a point that calls for further investigation, but at all events it will be noted that cancer of the cheek is not uncommon in Malays, and the fact that it is a little commoner in the women is suggestive. Unfortunately we have little or no information as to the habits of most of the cases which come to our attention.

*Malignant tumours of trophoblast*

It will be seen that the numbers of choriocarcinomas in the records are high and not confined to any one race. Llewellyn-Jones (1965), who has had charge of most of the cases referred to the Institute, has shown that trophoblastic tumours, including hydatidiform moles, occur at the rate of 1 in 600 pregnancies in the Kuala Lumpur hospitals, which is seven to ten times higher than the incidence in Europe or North America. We have included a record of the hydatidiform moles referred to the Institute in the three years under review, and it will be seen that the numbers are high in Chinese and Malays. Llewellyn-Jones has shown that they occur in all social classes but mostly in the lower, and at all ages in the child-bearing period, but above all in women over 40 years of age who have had many pregnancies.

SUMMARY

The foregoing is a review of the racial distribution of malignant tumours received in the Institute for Medical Research, Kuala Lumpur, in a three year period, 1961–63. The tumours, classified according to their anatomical sites, are set out in Table I so as to show their distribution in the three major races, Malays, Chinese and Indians. Three tumours occur with outstanding frequency, naso-pharyngeal cancers in Chinese, cancer of the cheek in Indians who chew betel nut, and trophoblastic tumours in all three races.

### REFERENCES

LLEWELLYN-JONES, D.—(1965) *J. Obstet. Gynaec. Br. Commonw.*, **72**, 242.
LOKE, W. Y.—(1965) *Br. J. Cancer*, **19**, 482.
MARSDEN, A. T. H.—(1958) *Br. J. Cancer*, **12**, 161.